# Glomerular Immune Deposition in MPO-ANCA Associated Glomerulonephritis Is Associated With Poor Renal Survival

**DOI:** 10.3389/fimmu.2021.625672

**Published:** 2021-03-25

**Authors:** Wei Lin, Chanjuan Shen, Yong Zhong, Joshua D. Ooi, Peter Eggenhuizen, Ya-Ou Zhou, Hui Luo, Jing Huang, Jin-Biao Chen, Ting Wu, Ting Meng, Zhou Xiao, Xiang Ao, Weisheng Peng, Rong Tang, Hongling Yin, Xiangcheng Xiao, Qiaoling Zhou, Ping Xiao

**Affiliations:** ^1^ Department of Pathology, Xiangya Hospital, Central South University, Changsha, China; ^2^ Department of Hematology, The Affiliated Zhuzhou Hospital Xiangya Medical College, Central South University, Zhuzhou, China; ^3^ Department of Nephrology, Xiangya Hospital, Central South University, Changsha, China; ^4^ Centre for Inflammatory Diseases, Monash University, Clayton, VIC, Australia; ^5^ Department of Rheumatology and Immunology, Xiangya Hospital, Central South University, Changsha, China; ^6^ Department of Medical Records and Information, Xiangya Hospital, Central South University, Changsha, China

**Keywords:** immune deposits, pauci-immune, ANCA, MPO – myeloperoxidase, AAV (ANCA-associated vasculitis)

## Abstract

**Background:**

Rapidly progressive glomerulonephritis caused by antineutrophil cytoplasmic antibody (ANCA)-associated vasculitis (AAV) is typically characterized as pauci-immune glomerulonephritis. However, immune complex (IC) deposition in the glomerulus has been reported in a growing number of studies. Here, we assess the presence of glomerular immune deposits alongside renal outcome in myeloperoxidase (MPO)-ANCA associated glomerulonephritis (MPO-ANCA GN).

**Methods:**

Clinical and histopathologic characteristics of 97 patients with MPO-ANCA GN classified by renal biopsy from January 2008 to December 2019 were extracted retrospectively from electronic medical records. The extent of immune deposits in the kidney (C3, C4, C1q, IgA, IgG, IgM) at diagnosis were analyzed by immunofluorescence (IF). Patients were followed up for a median period of 15 months. The response to treatment and outcomes of renal and histological lesion changes were also assessed.

**Results:**

In our study, 41% (40/97) of patients showed positive IF (≥2+) for at least one of the six immunoglobulin or complement components tested. Patients with IC deposits showed higher levels of serum creatinine (p=0.025), lower platelet counts (p=0.009), lower serum complement C3 (sC3) (≤790 ml/L) (p=0.013) and serum IgG (p=0.018) than patients with pauci-immune (PI) deposition at diagnosis. End-stage renal disease was negatively associated with eGFR (HR 0.885, 95% CI 0.837 to 0.935, p<0.0001), platelet count (HR 0.996, 95% CI 0.992 to 1.000, p=0.046) and serum globulin (HR 0.905, 95% CI 0.854 to 0.959, p=0.001). Patients with lower sC3 levels showed a worse renal outcome than the patients with normal sC3 at diagnosis (p=0.003). Analysis of the components of the renal deposits found that patients with IgG deposits exhibited a poorer renal outcome compared to patients that were IgG negative (p=0.028). Moreover, Bowman’s capsule rupture occurred less frequently in patients with IgM deposition compared with IgM negative counterparts (p=0.028). Vascular lesions and granuloma-like lesions had been seen more frequently in cases with IgA deposition than those without IgA deposition (p=0.03 and 0.015, respectively).

**Conclusion:**

In conclusion, patients with immune complex deposits in the kidney showed less platelet count, lower sC3 and sIgG levels, and higher serum creatinine levels. Patients with low sC3 at initial and with continued low sC3 during the treatment displayed a trend toward poorer kidney survival. Moreover, the IC group showed a worse renal outcome than the PI group, further enforcing the present strategy of introducing complement targeted therapies in AAV.

## Introduction

Antineutrophil cytoplasmic antibody (ANCA) associated glomerulonephritis (ANCA-GN) is typically characterized by no or little immune deposition in the glomerulus, which is defined as pauci-immune glomerulonephritis (GN) (1). However, glomerular immune-complex (IC) deposits were also reported in the renal biopsy of patients with ANCA-GN in some studies (2, 3). IC GN is characterized by granular deposits of polyclonal immunoglobulin (Ig) by immunofluorescence (IF) or immunohistochemistry (IHC), and complement is often co-deposited along with the Ig. Immune deposits were also found by electron microscopy in over half of the renal biopsies with ANCA-associated crescentic GN (2).

Analysis of the features of patients with ANCA-associated vasculitis (AAV) with immune complex deposition in the kidney showed more proteinuria, greater hypocomplementaemia, and greater glomerular hypercellularity (3, 4). Additionally, the type and location of the immune deposits often indicate the underlying etiology and display different histological lesions in ANCA-GN. Renal complement deposition and decreased serum complement levels observed in ANCA-GN patients indicated that ANCA-GN patients usually undergo complement activation (5, 6). Renal IgA deposition had also been reported in previous studies. Haas et al. and Chen et al. found that patients with necrotizing and/or crescentic GN with glomerular IgA deposits responded well to aggressive therapy (7, 8). Dudreuilh et al. reported that the appearance of IgG deposits in renal biopsy did not affect the renal outcome or probability of relapse (9).

Myeloperoxidase ANCA-associated vasculitis (MPO-AAV) often manifests as rapidly progressive GN (RPGN) and is much more common in China than PR3-AAV (10, 11). In this study, we focused on immune-complex deposits in renal biopsy specimens from MPO-AAV patients. Also, the clinical and histologic features and outcomes of patients were studied retrospectively.

## Methods

### Patients

The patients diagnosed with primary MPO-AAV and received a renal biopsy in Xiangya Hospital from January 2008 to December 2019 were enrolled in this study. All patients fulfilled 2012 revised International Chapel Hill Consensus Conference nomenclature of vasculitides. The clinical data and the pathological information of the biopsy were reviewed. Patients excluded from the study included those with anti-GBM antibody nephritis, lupus nephritis, IgA nephropathy, secondary vasculitis, such as drugs, infections. A total of 97 biopsies meeting these criteria were identified. The detail for recruitment was displayed in **Supplemental Figure 1**. The study protocol was in accordance with the ethical standards of the Ethics Committee of Xiangya Hospital of Central South University (IRB approval number 200901008) and with the Declaration of Helsinki. Informed consent was obtained from all individual participants included in the study.

### Clinical and Laboratory Findings

Patient age and gender were recorded. Vasculitis disease activity was evaluated by the Birmingham Vasculitis Activity Score (BVAS) (12). Laboratory data included the following: routine blood test, serum albumin, serum creatinine (Scr), erythrocyte sedimentation rate (ESR), 24 hours urine protein, C reactive protein (CRP), serum Complement 3 (sC3) and 4 (sC4), serum IgA (sIgA), serum IgG (sIgG) and serum IgM (sIgM) levels. All these parameters were collected at the approximate time of biopsy, and during follow-up. The estimated glomerular filtration rate (eGFR) was calculated according to the Chronic Kidney Disease Epidemiology Collaboration (CKD-EPI) equation (13).

### Renal Biopsy

All study patients received a renal biopsy when first hospitalized with newly-diagnosed MPO-ANCA GN, as well as before the commencement of immunosuppressive therapy. All 97 renal biopsies were reviewed using light microscopy by two pathologists who were blinded to the patient clinical data. The classification of MPO-ANCA GN was based on the Berden classification (14). The proportion of cellular crescent and global glomerulosclerosis, histological characteristics, including fibrinoid necrosis, Bowman’s capsule rupture, periglomerular inflammation, granuloma-like lesions, vascular lesions, thrombotic microangiopathy (TMA), and the scores of interstitial infiltrate and tubulointerstitial injury were all evaluated. Interstitial infiltrate and tubulointerstitial lesions were described as: mild (score of 1) for < 25% involvement, moderate (score of 2) for 25-50% involvement and severe (score of 3) for > 50% involvement (15).

### Immunofluorescence and Immunohistochemical

Glomerular immunofluorescence for IgA, IgM, IgG, C3, C1q, and C4 were scored by the intensity of immunostaining: negative (–), trace(±), mild (1+), moderate (2+), and strong (3+) (16). A biopsy with no or few immune deposits (pauci-immune) was defined as less than 2+ intensity of immunostaining (on an intensity scale 0, ±, 1+, 2+, 3+). The immune complex deposition was defined as a score of 2+ or higher in staining for any kind of immunoglobulin (Ig) and/or complement observed by immunofluorescence microscopy (2). Cases of positive for fibrin in immunofluorescence were calculated. Macrophages, neutrophils, and T cells infiltrated in renal tissue were assessed by probed with CD68, CD15, CD3 antibodies Macrophages, neutrophils, and T cells infiltrated in renal tissue were assessed by probed with CD68, CD15, CD3 antibodies in 51 cases of PI group and 39 cases of IC group, respectively. The frequencies of leukocytes cells were determined as the sum of expressing cells in all glomeruli divided by the number of glomerular cross-sections.

### Treatment and Follow-Up

As described previously, all 97 patients received the standard induction therapy including glucocorticoids combined with cyclophosphamide (17). Treatment resistance was defined as having an increasing and/or unchanged disease activity in patients with acute AAV after 4 weeks of treatment with standard induction therapy, or a reduction of 50% in BVAS after 6 weeks of treatment, or a chronic, persistent disease defined as the presence of at least 1 major or 3 minor items on the BVAS list after 12 weeks of therapy (18). End-stage renal disease (ESRD) was defined as eGFR < 15 mL/min/1.73 m^2^ and requiring permanent renal replacement therapy (RRT), or kidney transplantation. We assessed the response to the treatment and the development of ESRD during follow-up every 3 months. The patients were followed up until the death or the last follow-up date (June 30, 2020). All the patients were followed-up for a median period of 15 months, ranging from 1 month in those cases which died to 137 months.

### Statistical Analysis

Results are expressed as a percentage or mean ± SD for normally distributed variables and median (range) for non-normally distributed variables. All parameters were compared by the χ2 test or Fisher’s exact test for categorical data and Kruskal–Wallis test and Mann-Whitney U test for normally or non-normally distributed continuous data. Predictors of ESRD were evaluated in all patients using multivariate cox regression analysis, and the results are expressed as hazard ratios (HRs) with 95% confidence intervals (95% CIs) and p values. We evaluated baseline characteristics as potential covariates and transformed these to proper function forms to fit the models best and meet the inferential goal of finding predictors for the outcome of ESRD. Hemoglobin, blood platelet count, serum creatine, ESR, serum C3, C4 and serum immunoglobulin, and tubulointerstitial scores were used as continuous variables. Age, eGFR, CRP, and urine protein indexes were categorized as quartiles. The percentage of different histological classification were used as categorical covariates and changed contrast by using indicator method. The presence of IC deposits (≥2+) and gender, histological characteristics (fibrinoid necrosis, and Bowman’s capsule rupture, TMA, periglomerular inflammatory, granulomatous lesions) on biopsy were used as binary covariates. Each regression model was conducted with a selection criterion of P<0.05. Then, significant predictor variables were chosen by backward elimination using anαlevel of 0.05 until all variables remaining in the model show significant association with ESRD. Renal survival and patient survival were evaluated by Kaplan-Meier curves using log-rank and generalized Wilcoxon-Breslow tests. A *P*-value<0.05 was considered to be statistically significant. Analyses were performed using SPSS statistical software (version 23.0).

## Results

### Baseline Patient Characteristics

Among all 97 patients with MPO-ANCA GN, 59% (57/97) of patients had no or few deposits in their glomeruli from renal biopsy, which were classified as the “pauci-immune” (PI) group, while the other 41% (40/97) of cases were classified as the “immune complex” (IC) group. The clinical and histopathological characteristics of the two groups are shown in **Table 1**.

**Table 1:** Comparison of clinicopathologic parameters between MPO-ANCA-GN patients with and without immune complex deposits.

	PI*^a^* group (*N = 57*)	IC*^b^* group (*N = 40*)	Total (*N = 97*)	*P-*value
Age (year)	58 (51.50, 65.00)	58 (45.33, 65.00)	58 (49.5, 65.0)	0.424
Sex (male/female)	26/31	27/13	53/44	0.033*
Hemoglobin (g/dl) (mean, SD)	78.28 ± 15.43	81.10 ± 19.22	79.44 ± 17.05	0.426
Platelet (×10^3^/mm3) (mean, SD)	276.02 ± 95.43	224.15 ± 93.48	254.63 ± 97.58	0.009*
Serum albumin (g/L) (mean, SD)	44.02 ± 17.29	43.60 ± 16.39	43.85 ± 16.84	0.903
Serum globulin (g/L) (mean, SD)	31.78 ± 7.06	31.58 ± 6.13	31.69 ± 6.66	0.885
Blood urea nitrogen (mmol/L) (median, IQR)	14.13 (9.51, 21.33)	18.29 (9.77, 23.55)	15.99 (9.51, 21.79)	0.160
Serum creatinine (mg/dl) (median, IQR)	3.33 (2.38, 5.51)	4.63 (2.85, 8.26)	4.08 (2.45, 6.19)	0.025*
eGFR*^c^* (ml/min per 1.73 m2) (median, IQR)	15.14 (8.55, 25.70)	11.04 (6.31, 22.34)	13.88 (7.43, 24.94)	0.079
Urinary protein (g/24 h) (median, IQR)	1.28 (0.48, 3.05)	1.24 (0.58, 2.98)	1.28 (0.49, 3.00)	0.849
Urinary blood cell count (/UL) (median, IQR)	300.00 (60.50, 496.08)	283.95 (91.4, 580.9)	300.00 (85.25, 513.43)	0.819
CRP*^d^*, mg/dL (media, IQR)	24.15 (8.76, 71.15)	7.97 (2.91, 22.9)	16.05 (5.05, 42.85)	0.001*
ESR*^e^* (mm/h) (mean, SD)	76.77 ± 34.27	60.30 ± 39.81	70.22 ± 37.26	0.036*
MPO-ANCA*^f^* titer (U/ml) (mean, SD)	95.01 ± 42.89	83.92 ± 45.08	90.16 ± 43.86	0.319
Serum immunological indexes				
sC3 (mg/L) (mean, SD)	829.66 ± 248.23	699.62 ± 241.18	776.27 ± 252.40	0.013*
sC4 (mg/L) (mean, SD)	245.61 ± 99.32	259.09 ± 128.35	251.14 ± 111.69	0.565
sIgA (mg/L) (median, IQR)	2655.0 (1790.0, 3692.5)	1790.0 (1462.5, 2972.5)	2490.0 (1550.0, 3180.0)	0.023*
sIgM (mg/L) (median, IQR)	978.5 (748.7, 1540.0)	982.0 (754.5, 1220.0)	979 (754.0, 1520.0)	0.736
sIgG (g/L) (mean, SD)	15.40 ± 4.85	12.97 ± 4.85	14.40 ± 4.97	0.018*
Classfication, n%				
Focal	5, 8.77%	7, 17.95%	12, 12.50%	0.096
Mixed	27, 47.37%	9, 23.08%	36, 37.50%	
Crescentic	14, 24.56%	12, 30.77%	26, 27.08%	
Sclerotic	11, 19.30%	11, 28.21%	22, 22.92%	
Histological characteristics				
Fibrinoid necrosis, n%	29, 50.88%	19, 47.50%	48, 49.48%	0.837
Bowman’s capsule rupture, n%	25, 43.86%	10, 25%	35, 36.08%	0.085
Periglomerular inflammatory, n%	19, 33.33%	8, 20%	27, 27.84%	0.173
Granulomatous lesions, n%	5, 8.77%	7, 17.50%	12, 12.37%	0.224
TMA*^g^*, n%	13, 22.81%	13, 32.50%	26, 26.80%	0.354
Interstitial infiltrates (n, 0/1/2/3)	0/23/29/5	0/20/14/6	0/43/43/11	0.580
Tubulointerstitial lesions (n, 0/1/2/3)	0/24/27/6	0/14/19/7	0/38/46/13	0.215
Positive of fibrin, n%	17, 29.82%	18, 45.00%	35, 36.08%	0.139
BVAS*^h^*	16.98 ± 6.42	18.23 ± 5.39	17.49 ± 6.02	0.319
RPGN^i^, n %	14, 24.56%	16, 40.00%	30, 30.92%	0.082
Treatment-resistant to the treatment, n%	12, 26.09%	11, 42.31%	23, 31.94%	0.156

^a^PI, pauci-immune; ^b^IC, immune complex; ^c^eGFR, estimated glomerular filtration rate; ^d^CRP, C-reactive protein; ^e^ESR, Erythrocyte Sedimentation Rate; ^f^MPO-ANCA, myeloperoxidase anti-neutrophil cytoplasmic antibody; ^g^TMA, thrombotic microangiopathy; ^h^BVAS, Birmingham vasculitis activity score; ^i^RPGN, rapidly progressive glomerulonephritis. *P < 0.05.

Immune complex deposits in the glomeruli were more frequently found in males than females with MPO-ANCA GN. Patients with immune complex deposits in the kidney showed less platelet count, lower sC3, sIgA and sIgG level, and higher serum creatinine levels than the PI group (p=0.009, 0.013, 0.018, 0.023, 0.025, respectively). CRP and ESR levels were higher in the PI group compared with the IC group (p=0.001 and 0.036, respectively). It is noteworthy that we also found more glomerular fibrin deposition in patients in the IC group than in the PI group, although the differences observed did not reach statistical significance (p=0.139). We also assessed the frequency of neutrophils, macrophages and T cells in the glomerular, periglomerular and interstitial compartments by immunostaining. The immune cells were detected in similar numbers in PI and IC groups as shown in **Table 1**.

### Risk Factors for ESRD

Of the 97 patients, ESRD occurred in 42 (43.30%) patients during follow-up. Furthermore, 55% (22/40) patients in IC group and 35.09% (20/57) patients in PI group experienced ESRD during follow-up. In the multivariate analysis (**Table 2**), ESRD was shown to be negatively associated with eGFR (HR 0.885, 95% CI 0.837 to 0.935, p<0.0001), platelet count (HR 0.996, 95% CI 0.992 to 1.000, p=0.046) and serum globulin (HR 0.905, 95% CI 0.854 to 0.959, p=0.001).

**Table 2:** Multivariable predictors of ESRD*^a^* by multivariate COX regression analysis.

Predictor	*P-*value	HR*^b^* (95% CI*^c^*)
Platelet (×10^3^/mm^3^)	0.046	0.996 (0.992,1.000)
Serum globulin (g/L)	0.001	0.905 (0.854, 0.959)
eGFR	<0.0001	0.885 (0.837, 0.935)
sIgG	0.051	0.917 (0.837, 1.000)
Interstitial infiltrates	0.065	1.529 (0.974, 2.400)

^a^ESRD, end stage renal disease; ^b^HR, hazard ratio; ^c^CI, confidence interval.

### Renal Histopathology and IC Deposits

As shown in **Table 3**, C3 was the most common complement component found in the glomeruli of kidney biopsy specimens. C3 IF staining of 1+ was seen in 18 (18.56%) cases, while there were only 19 cases (19.59%) that were more intensively stained. C1q was also seen in 10 (10.75%) renal biopsies with IF staining ≥1+, but only 4 (4.30%) cases showed stronger staining (IF≥2+). Only 7 patients (13.73%) showed a weak stain (1+) in C4 deposits. For renal immunoglobulin deposition, IgA, IgM and IgG showed an IF intensity of 1+ in 18.75%, 17.71%, 13.54% cases, respectively and strong intensity IF (≥2+) was found in 5.21%, 28.12%, 18.75% cases, respectively (**Table 3**).

**Table 3:** Immunofluorescence findings in biopsies and histopathological features in MPO-ANCA-GN.

		The proportion of crescentic glomeruli, %			Fibrinoid necrosis		Bowman’s capsule Rupture		Periglomerular inflammation		Granulomatous lesions		Vascular lesions		Thrombotic microangiopathy		Proportion of sclerotic glomeruli, %		Interstitial infiltrates (score≥2)		Interstitial fibrosis/tubular atrophy lesions (score≥2)	
		n (%)	Mean ± STD	*P*	n (%)	*P*	n (%)	*P*	n (%)	*P*	n (%)	*P*	n (%)	*P*	n (%)	*P*	Mean ± SD	*P*	n (%)	*P*	n (%)	*P*
C3	Number of negative	60 (61.86)	39.98 ± 26.05	0.788	29 (48.33)	0.492	27 (45.00)	0.066	20 (33.33)	0.278	7 (11.67)	0.831	18 (30.00)	0.496	16 (26.67)	0.813	32.84 ± 22.98	0.939	33 (55.00)	0.491	35 (58.33)	0.735
	Number of 1+	18 (18.55)	43.77 ± 23.48		11 (61.11)		4 (22.22)		4 (22.22)		3 (16.67)		7 (38.89)		4 (22.22)		32.35 ± 27.27		12 (66.67)		11 (61.11)	
	Number ≥2+	19 (19.59)	37.93 ± 25.83		8 (42.11)		4 (21.05)		3 (15.79)		2 (10.53)		4 (21.05)		6 (31.58)		39.50 ± 37.31		9 (47.37)		13 (68.42)	
C4	Number negative	44 (86.27)	44.21 ± 27.31	0.805	21 (47.73)	0.703	20 (45.45)	0.393	16 (36.36)	0.401	8 (18.18)	0.797	18 (40.91)	0.690	16 (36.36)	1.000	35.21 ± 25.18	0.132	28 (63.64)	0.411	25 (56.82)	1.000
	Number of 1+	7 (13.73)	45.75 ± 17.67		4 (57.14)		2 (28.57)		1 (14.29)		1 (14.29)		2 (28.57)		3 (42.86)		19.64 ± 12.30		3 (42.86)		4 (57.14)	
C1q	Number of negative	83 (89.25)	41.39 ± 25.06	0.351	42 (50.60)	0.370	33 (39.76)	0.087	27 (32.53)	0.032*	12 (14.46)	0.430	24 (28.92)	0.318	24 (28.92)	0.825	33.02 ± 26.04	0.138	49 (59.04)	0.835	49 (59.04)	0.735
	Number of 1+	6 (6.45)	26.46 ± 29.69		1 (16.67)		0 (0)		0 (0)		0 (0)		2 (33.33)		1 (16.67)		47.87 ± 34.24		3 (50)		3 (50)	
	Number ≥2+	4 (4.30)	34.14 ± 33.45		2 (50)		1 (25)		0 (0)		0 (0)		3 (75)		1 (25)		54.32 ± 34.79		2 (50)		4 (100)	
IgA	Number of negative	73 (76.04)	40.83 ± 24.70	0.272	36 (49.32)	0.917	31 (42.47)	0.089	24 (32.88)	0.032*	8 (10.96)	0.015*	22 (30.14)	0.03*	19 (26.03)	0.757	32.82 ± 25.96	0.685	42 (57.53)	0.862	43 (58.90)	0.858
	Number of 1+	18 (18.75)	42.54 ± 24.86		9 (50.00)		3 (16.67)		1 (5.56)		1 (5.56)		3 (16.67)		5 (27.78)		40.69 ± 30.40		9 (50.00)		12 (66.67)	
	Number ≥2+	5 (5.21)	20.45 ± 40.91		2 (40.00)		1 (20.00)		2 (40.00)		3 (60.00)		4 (80.00)		2 (40.00)		34.22 ± 31.00		3 (60.00)		3 (60.00)	
IgM	Numberof negative	52 (54.17)	26.71 ± 43.22	0.022*	31 (59.62)	0.075	25 (48.08)	0.028*	20 (38.46)	0.034*	7 (13.46)	1.000	14 (26.92)	0.741	13 (25.00)	0.700	27.00 ± 35.28	0.454	32 (61.54)	0.155	30 (57.69)	0.432
	Number of 1+	17 (17.71)	19.55 ± 28.86		6 (35.29)		5 (29.41)		4 (23.53)		2 (11.76)		6 (35.29)		6 (35.29)		22.01 ± 38.82		11 (64.71)		9 (52.94)	
	Number ≥2+	27 (28.12)	23.51 ± 40.30		10 (37.04)		5 (18.52)		3 (11.11)		3 (11.11)		9 (33.33)		7 (25.93)		29.67 ± 34.37		11 (40.74)		19 (70.37)	
IgG	Number of negative	65 (67.71)	40.98 ± 24.93	0.786	30 (46.15)	0.725	24 (36.92)	0.571	18 (27.69)	0.613	7 (10.77)	0.457	18 (27.69)	0.711	14 (21.54)	0.185	31.03 ± 24.27	0.342	36 (55.38)	0.550	37 (56.92)	0.242
	Number of 1+	13 (13.54)	36.81 ± 30.74		7 (53.85)		6 (46.15)		5 (38.46)		1 (7.69)		5 (38.46)		5 (38.46)		36.16 ± 24.99		9 (69.23)		7 (53.85)	
	Number ≥2+	18 (18.75)	40.39 ± 24.82		10 (55.56)		5 (27.78)		4 (22.22)		4 (22.22)		6 (33.33)		7 (38.89)		44.94 ± 34.86		9 (50.00)		14 (77.78)	

*P < 0.05, differences in three groups were compared by analysis of the Kruskal-Wallis test or Fisher’s Exact Test.

As shown in **Table 3**, Bowman’s capsule rupture (p=0.028) and periglomerular inflammation (p=0.034) were less commonly seen in biopsies with IgM deposition.

Besides, in the renal biopsies with stronger IgA deposition (IF≥2+), granuloma-like lesions and vascular lesions were more frequently seen than those without IgA deposition (p=0.03 and 0.015, respectively).

### Renal Survival Analysis

**Figure 1:**
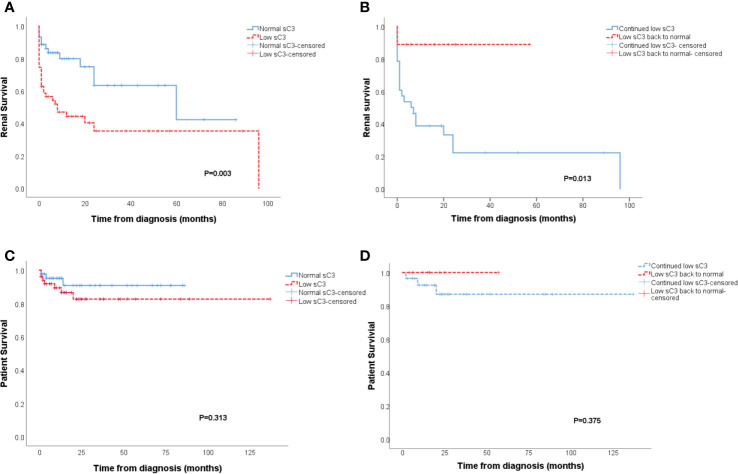
Patients with low sC3 showed worse renal outcomes than patients with normal sC3 level at diagnosis (p = 0.003) **(A)**. Patients who persist with low sC3 exhibit a substantially higher degree of ESRD than the patients whose sC3 level returned to normal during the treatment process (p = 0.013) **(B)**. The patient survival of patients with low sC3 at diagnosis and persists with low sC3 in the treatment **(C, D)**.

Analysis of renal survival showed patients which had lower sC3 levels had higher rates of ESRD compared to cases with normal serum C3 values (p=0.003, **Figure 1A**). Furthermore, the patients with continued low sC3 during therapy showed worse renal outcomes than the patients with sC3 levels returning to normal during treatment (p=0.013, **Figure 1B**). Also, patients with low sC3 both at initial and with continued low sC3 during the treatment all displayed a trend toward decreased patient survival (**Figures 1C, D**) without reaching statistical significance.

**Figure 2:**
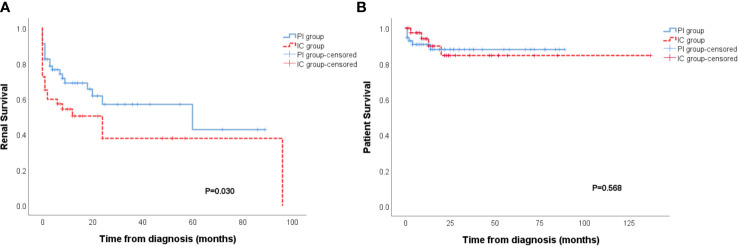
Patients of the immune complex deposits (IC) group had a higher incidence of ESRD than the pauci-immune (PI) group (p = 0.030) **(A)**. No distinction of patient survival had been observed between PI and IC group **(B)**.

**Figure 3:**
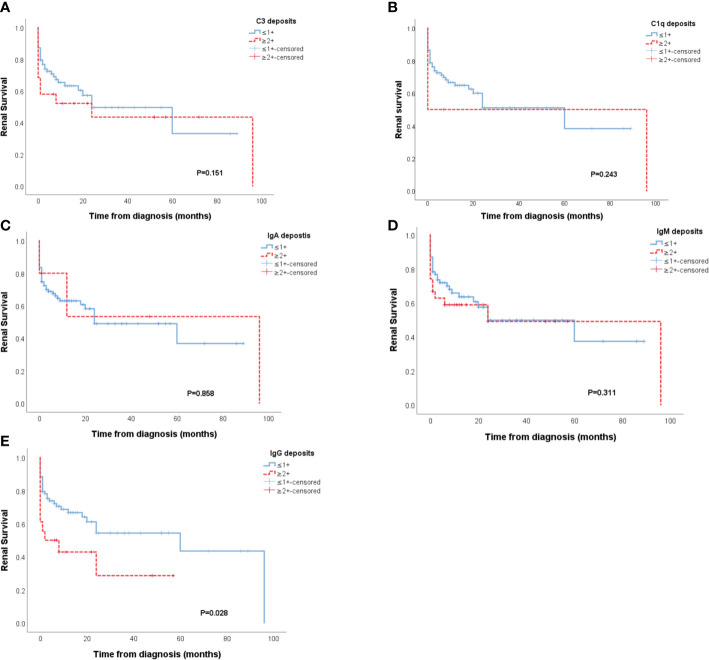
Renal survival of patients with and without C3, C1q, IgA, IgM deposits **(A–D)**. Patients with IgG deposits showed poorer renal prognosis than patients that had no or low IgG deposits in the glomeruli (p = 0.028) **(E)**.

The IC group showed a worse renal outcome than the PI group (Breslow p=0.030) (**Figure 2A**), but there was no difference in patient survival (**Figure 2B**). In further analysis of the outcome of patients with different types of immune deposits, no differences in renal survival were observed between patients with kidney IF intensities ≥2+ for C3, C1q, IgA, IgM and patients with kidney IF intensities ≤1+ for C3, C1q, IgA, IgM (**Figures 3A–D**). However, patients with IgG deposits (≥2+ in IF) showed a poorer renal outcome than the patients with no or low level deposits (≤1+ in IF) (p=0.028) (**Figure 3E**).

## Discussion

Immune complex deposition in MPO-AAV does not seem to be as uncommon as previously considered. We found that 41% of cases had at least one Ig or complement component deposited in the glomeruli (IF≥2+ on a scale of 0 to 4). Though the absence or paucity of IC localization in the glomeruli is classically defined as a characteristic of ANCA-GN, similar to our study, there have been some previous studies that reported ANCA-GN as not always being pauci-immune. Hass et al. reported that 54% of 126 cases showed immune complex deposits by electron microscopy (2). Another study also showed 26.4% of ANCA-GN cases exhibited immunostaining of ≥2+ intensity in renal biopsies (19). The prevalence of stronger IF findings in our study was higher than previously reported, which may be ascribed to recruiting only MPO-ANCA GN patients rather than all the ANCA-GN patients including PR3-ANCA-GN, and there were no comparative data in detail about IC deposition between MPO-ANCA GN and PR3-ANCA-GN.

In our study, the serological tests at diagnosis, namely lower platelet count, sC3, sIgG, and higher sCr can be seen in patients with IC deposits compared with the PI group. The most plausible reason for that sCr is significantly higher in the patients with IC deposits but eGFR is not significantly different between the two groups is that there were more males in the IC group compared with PI group. Low levels of platelets suggest active coagulation particularly the renal deposition of fibrin which has been shown to a prominent mediator of inflammatory kidney injury (20). Although there was a trend of increased glomerular fibrin deposition in MPO-AAV-GN patients with immune complex deposits in the kidney, this did not reach statistical significance. Further studies with a large number of patients are needed to quantitate the level of renal fibrin deposition in the kidney of MPO-ANCA-GN patients, which may suggest the renal presence of another important potential therapeutic target.

Meaningfully, platelet counts, serum globulin and eGFR were shown to be risk factors for ESRD in our multifactorial analysis. In line with previous reports, patients with lower sC3 at diagnosis displayed poorer renal survival (6). In addition, treatment resistance was observed to be negatively associated with platelet count and sC3 level in our previous study (21). Our data here is consistent with the findings of others (15, 22–26). We found that there was a significantly poorer renal outcome in patients with persistently lower sC3 levels than those whose sC3 levels recovered to normal during therapy upon follow-up. The documented role of sC3 reduction in treatment resistance and renal prognosis in the MPO-AAV patients, as shown in our data, support the use of complement targeted therapies in AAV, such as the C5a receptor inhibitor CCX168 (21, 27–30).

However, we did not observe significant differences in BVAS. The Berden classification of renal injury in ANCA-GN is widely accepted (14). This classification does allow the intensity of immune inflammation and damage to be assessed. Nonetheless, we find no differences of the four classes of MPO-ANCA-GN, active and chronic lesions between PI and IC groups. The reason for this is unclear. We suspect that one plausible explanation for this is the small number of patients in the present study. It is now widely accepted that cell mediated immunity is prominent in MPO-AAV patients and has been shown to be a major components in inducing inflammatory injury in many models of this disease (31). The immune cells were detected in similar numbers in the glomerular, periglomerular and interstitial compartments in PI and IC groups (**Table S1**). Furthermore, ESR and CPR levels, which are routinely used as biomarkers of disease activity in clinical work, were lower in the group with IC than the pauci-immune group. This data is consistent with that obtained by other investigators (32, 33), but our data were in contrast to the study by Brons et al. showing immune deposits in skin lesions of patients with Wegener’s granulomatosis during active disease (34). The exact reason for this remains unknown, which might be associated with the production and deposition of ICs in the kidney in MPO-AAV patients. It suggested that IC in the glomeruli might have no relation to systemic disease activity. On the contrary, IC in the glomeruli might be associated with the chronic process in MPO-ANCA GN.

Moreover, different types of deposition also have various implications in MPO-ANCA GN. In agreement with the results of previous studies, the deposition of complement in our study has suggested again that complement activation usually occurs in MPO-ANCA GN. Gou et al. detected the alternative complement pathway activation fragment Bb deposited both in the kidney and in urine which confirmed complement activation through the alternative pathway that occurred in the development of AAV (35). In our study, more commonly C3 and less commonly C1q had been seen deposited in the kidney, similar to previous reports (36), suggesting that the complement system may be commonly activated by the alternative pathway but also by the classical complement pathway less often (5). However, contrary to previous studies (29, 36), both C3 and C1q deposition in the kidney didn’t show a special connection with any histopathological signs of MPO-ANCA-GN.

The findings of immunoglobulin in kidney specimens were accompanied by C3 deposition. IgG deposits had no relation with histological changes, but it was associated with a higher risk for ESRD. The patients with IgG deposition demonstrated a higher rate of ESRD, which was in contrast to another retrospective study (9). The basis for this discrepancy needs to be investigated in future studies. Differences in participants and an insufficient number of cases in both studies may partly explain this discrepancy. Different from IgG deposits, IgA seems to be more related to histological characteristics of ANCA-GN. Granuloma-like and vascular lesions had been more frequently seen in the cases with IgA deposition. As reported previously, IgA nephropathy (IgAN) with ANCA positivity showed less typical mild, focal, and segmental mesangial and endocapillary hypercellularity and more severe clinical features than the cases that were ANCA negative (7, 37). Also, some reports proposed that patients both with IgA deposition and ANCA positivity responded well to aggressive immunosuppressive therapy (37, 38). But, there were few studies about ANCA-GN with IgA deposition (32). In our study, there was no difference in renal outcome between patients with and without IgA deposition. However, granuloma-like and vascular lesions, as the characteristic pathological changes of MPO-ANCA GN, were closely associated with IgA deposition.

Unexpectedly, on histological sections, we also discovered that Bowman’s capsule rupture and periglomerular inflammation occurred infrequently in the cases with IgA and IgM deposits. Bowman’s capsule is implicated in functionally isolating potential immune effectors thereby preventing injury to the glomerulus. So, the rupture was considered as a trigger of inflammatory cell migration (such as CD8+ T cells and macrophagocytes) and crescentic formation (39). The inverse negative relationship between IC deposits and Bowman’s capsule rupture and periglomerular inflammation suggests that Bowman’s capsule rupture and periglomerular inflammation with subsequent inflammatory cell infiltration into the glomerulus might be related to the clearance of IC in MPO-ANCA GN. This needs more clinical observation and animal studies to further confirm.

Our study has several limitations as it is retrospective and has a small case load, which may have introduced information and recall bias. Moreover, the number of glomeruli in some biopsies is relatively small, which might lead to some bias and reduce the power to demonstrate associations between histopathological findings and immune deposits. The relatively short period of follow-up was another limitation in our study, which also influenced the analysis of the prognosis of patients.

In conclusion, patients with immune complex deposits in the kidney showed less platelet count, lower sC3 and sIgG levels and higher serum creatinine levels. Patients with low sC3 at initial and with continued low sC3 during treatment all displayed a trend toward poorer kidney survival. Moreover, the IC group showed worse renal outcome than the PI group, further enforcing the present orientation to introduce complement targeted therapies in AAV. Also, patients with IgG deposits showed a poorer renal outcome, which can help to evaluate prognosis to some extent. IgA deposits were associated with vascular and granuloma-like lesions in MPO-ANCA GN, suggesting the connection between the immune deposits and histological features in ANCA-GN. Our findings suggest MPO-AAV-associated renal damage can be immune complex-induced and could assist in guiding clinical work.

## Data Availability Statement

The raw data supporting the conclusions of this article will be made available by the authors, without undue reservation.

## Ethics Statement

The studies involving human participants were reviewed and approved by the Ethics Committee of Xiangya Hospital of Central South University (IRB approval number 200901008). The patients/participants provided their written informed consent to participate in this study.

## Author Contributions

YZ, PX, XX, HL, and QZ contributed to the conception of the study. WL, YZ, and JO performed the analysis with constructive discussions. CS, J-BC, and XA analyzed and interpreted the data. HY, WL, and ZX completed the pathological analysis. RT, TW, WP, JH, and Y-OZ contributed significantly to the clinical follow-up. WL finished the manuscript. YZ and JO supervised and edited the manuscript. PE edited the manuscript. All authors contributed to the article and approved the submitted version.

## Funding

This work was supported by the National Natural Science Foundation of China (81800641), the Natural Science Foundation of Hunan Province (2018JJ3853; 2018JJ3818), the Project of Health Commission of Hunan province (C2019184), Chinese Society of Nephrology (18020010780), and the Elite Program of Xiangya hospital.

## Conflict of Interest

The authors declare that the research was conducted in the absence of any commercial or financial relationships that could be construed as a potential conflict of interest.

The reviewer SH declared a shared affiliation with several of the authors, JO and PE, to the handling editor at the time of review.

## References

[1] Jennette JC Falk RJ Bacon PA Basu N Cid MC Ferrario F. 2012 revised international chapel hill consensus conference nomenclature of vasculitides. Arthritis Rheumatol (2013) 65:1–11. 10.1002/art.37715 23045170

[2] Haas M Eustace JA. Immune complex deposits in ANCA-associated crescentic glomerulonephritis: A study of 126 cases. Kidney Int (2004) 65:2145–52. 10.1111/j.1523-1755.2004.00632.x 15149327

[3] Neumann I Regele H Kain R Birck R Meisl FT. Glomerular immune deposits are associated with increased proteinuria in patients with ANCA-associated crescentic nephritis. Nephrol Dial Transpl (2003) 18:524–31. 10.1093/ndt/18.3.524 12584274

[4] Yu F Chen M Wang SX Zou WZ Zhao MH Wang HY. Clinical and pathological characteristics and outcomes of Chinese patients with primary anti-neutrophil cytoplasmic antibodies-associated systemic vasculitis with immune complex deposition in kidney. Nephrol (Carlton) (2007) 12:74–80. 10.1111/j.1440-1797.2006.00713.x 17295665

[5] Xing GQ Chen M Liu G Heeringa P Zhang JJ Zheng X. Complement activation is involved in renal damage in human antineutrophil cytoplasmic autoantibody associated pauci-immune vasculitis. J Clin Immunol (2009) 29:282–91. 10.1007/s10875-008-9268-2 19067130

[6] Choi H Kim Y Jung SM Song JJ Park YB Lee SW. Low serum complement 3 level is associated with severe ANCA-associated vasculitis at diagnosis. Clin Exp Nephrol (2019) 23:223–30. 10.1007/s10157-018-1634-7 30168048

[7] Haas M Jafri J Bartosh SM Karp SL Adler SG Meehan SM. Anca-associated crescentic glomerulonephritis with mesangial IgA deposits. Am J Kidney Dis (2000) 36:709–18. 10.1053/ajkd.2000.17615 11007672

[8] Chen T Xia E Chen T Zeng C Liang S Xu F. Identification and external validation of IgA nephropathy patients benefiting from immunosuppression therapy. EBioMedicine (2020) 52:102657. 10.1016/j.ebiom.2020.102657 32062356 PMC7016365

[9] Dudreuilh C Fakhouri F Vigneau C Augusto JF Machet MC Rabot N. The presence of renal igg deposits in necrotizing crescentic glomerulonephritis associated with ANCA is not related to worse renal clinical outcomes. Kidney Dis (Basel) (2020) 6:98–108. 10.1159/000503969 32309292 PMC7154286

[10] Chen M Yu F Zhang Y Zhao MH. Clinical [corrected] and pathological characteristics of Chinese patients with antineutrophil cytoplasmic autoantibody associated systemic vasculitides: A study of 426 patients from a single center. Postgrad Med J (2005) 81:723–7. 10.1136/pgmj.2005.034215 PMC1743382 16272238

[11] Hong Y Shi P Liu X Yang L Li K Xu F. Distinction between MPO-ANCA and PR3-ANCA-associated glomerulonephritis in Chinese patients: A retrospective single-center study. Clin Rheumatol (2019) 38:1665–73. 10.1007/s10067-019-04458-9 30737591

[12] Mukhtyar C Lee R Brown D Carruthers D Dasgupta B Dubey S. Modification and validation of the Birmingham vasculitis activity score (version 3). Ann Rheum Dis (2009) 68:1827–32. 10.1136/ard.2008.101279 19054820

[13] Levey AS Stevens LA Schmid CH Zhang YL Castro AF 3rd, Feldman HI. A new equation to estimate glomerular filtration rate. Ann Intern Med (2009) 150:604–12. 10.7326/0003-4819-150-9-200905050-00006 PMC2763564 19414839

[14] Berden AE Ferrario F Hagen EC Jayne DR Jennette JC Joh K. Histopathologic classification of ANCA-associated glomerulonephritis. J Am Soc Nephrol (2010) 21:1628–36. 10.1681/asn.2010050477 20616173

[15] Chen Y Bao H Liu Z Liu X Gao E Zeng C. Risk factors for renal survival in Chinese patients with myeloperoxidase-ANCA-associated GN. Clin J Am Soc Nephrol (2017) 12:417–25. 10.2215/cjn.06200616 PMC5338707 28148558

[16] Sethi S Haas M Markowitz GS D’Agati VD Rennke HG Jennette JC. Mayo clinic/renal pathology society consensus report on pathologic classification, diagnosis, and reporting of gn. J Am Soc Nephrol (2016) 27:1278–87. 10.1681/asn.2015060612 PMC4849835 26567243

[17] Huang L Zhong Y Ooi JD Zhou YO Zuo X Luo H. The effect of pulse methylprednisolone induction therapy in Chinese patients with dialysis-dependent MPO-ANCA associated vasculitis. Int Immunopharmacol (2019) 76:105883. 10.1016/j.intimp.2019.105883 31536905

[18] Hellmich B Flossmann O Gross WL Bacon P Cohen-Tervaert JW Guillevin L. Eular recommendations for conducting clinical studies and/or clinical trials in systemic vasculitis: Focus on anti-neutrophil cytoplasm antibody-associated vasculitis. Ann Rheum Dis (2007) 66:605–17. 10.1136/ard.2006.062711 PMC2703775 17170053

[19] Scaglioni V Scolnik M Catoggio LJ Christiansen SB Varela CF Greloni G. Anca-associated pauci-immune glomerulonephritis: Always pauci-immune? Clin Exp Rheumatol (2017) 35 Suppl;103:55–8. 10.32388/df1ec8 28229825

[20] Fujita T Yamabe H Shimada M Murakami R Kumasaka R Nakamura N. Thrombin enhances the production of monocyte chemoattractant protein-1 and macrophage inflammatory protein-2 in cultured rat glomerular epithelial cells. Nephrol Dial Transpl (2008) 23:3412–7. 10.1093/ndt/gfn352 18622025

[21] Huang L Shen C Zhong Y Ooi JD Zhou YO Chen JB. Risk factors for treatment resistance and relapse of Chinese patients with MPO-ANCA-associated vasculitis. Clin Exp Med (2020) 20:199–206. 10.1007/s10238-020-00614-7 32078076

[22] Manenti L Vaglio A Gnappi E Maggiore U Allegri L Allinovi M. Association of serum c3 concentration and histologic signs of thrombotic microangiopathy with outcomes among patients with ANCA-associated renal vasculitis. Clin J Am Soc Nephrol (2015) 10:2143–51. 10.2215/cjn.00120115 PMC4670755 26542163

[23] García L Pena CE Maldonado R Costi C Mamberti M Martins E. Increased renal damage in hypocomplementemic patients with ANCA-associated vasculitis: Retrospective cohort study. Clin Rheumatol (2019) 38:2819–24. 10.1007/s10067-019-04636-9 31222573

[24] Deshayes S Aouba A Khoy K Mariotte D Lobbedez T Martin Silva N. Hypocomplementemia is associated with worse renal survival in anca-positive granulomatosis with polyangiitis and microscopic polyangiitis. PLoS One (2018) 13:e0195680. 10.1371/journal.pone.0195680 29621352 PMC5886583

[25] Crnogorac M Horvatic I Kacinari P Ljubanovic DG Galesic K. Serum c3 complement levels in anca associated vasculitis at diagnosis is a predictor of patient and renal outcome. J Nephrol (2018) 31:257–62. 10.1007/s40620-017-0445-3 29027625

[26] Chen Z Lin L Yang W Chen N Lin Y. Clinical characteristics and prognostic risk factors of anti-neutrophil cytoplasmic antibody (anca)-associated vasculitides (aav). Int Immunopharmacol (2020) 87:106819. 10.1016/j.intimp.2020.106819 32717565

[27] Kallenberg CG Heeringa P. Complement is crucial in the pathogenesis of ANCA-associated vasculitis. Kidney Int (2013) 83:16–8. 10.1038/ki.2012.371 23271485

[28] Chen M Jayne DRW Zhao MH. Complement in ANCA-associated vasculitis: Mechanisms and implications for management. Nat Rev Nephrol (2017) 13:359–67. 10.1038/nrneph.2017.37 28316335

[29] Hilhorst M van Paassen P van Rie H Bijnens N Heerings-Rewinkel P van Breda Vriesman P. Complement in ANCA-associated glomerulonephritis. Nephrol Dial Transpl (2017) 32:1302–13. 10.1093/ndt/gfv288 26275893

[30] Brilland B Garnier AS Chevailler A Jeannin P Subra JF Augusto JF. Complement alternative pathway in ANCA-associated vasculitis: Two decades from bench to bedside. Autoimmun Rev (2020) 19:102424. 10.1016/j.autrev.2019.102424 31734405

[31] Kitching AR Anders HJ Basu N Brouwer E Gordon J Jayne DR. Anca-associated vasculitis. Nat Rev Dis Primers (2020) 6:71. 10.1038/s41572-020-0204-y 32855422

[32] Ma Y Chen L Xu Y Han Q Yu B Zhao J. The clinicopathologic characteristics and complement activation of antineutrophil cytoplasmic antibody-associated vasculitides with glomerular IgA deposition. Appl Immunohistochem Mol Morphol (2019) 28:e87–93. 10.1097/pai.0000000000000819 31789820

[33] Sumida K Ubara Y Nomura K Hoshino J Suwabe T Hiramatsu R. Anca-associated crescentic glomerulonephritis with immune complex deposits. Clin Nephrol (2012) 77:454–60. 10.5414/cn107254 22595387

[34] Brons RH de Jong MC de Boer NK Stegeman CA Kallenberg CG Tervaert JW. Detection of immune deposits in skin lesions of patients with Wegener’s granulomatosis. Ann Rheum Dis (2001) 60:1097–102. 10.1136/ard.60.12.1097 PMC1753448 11709450

[35] Gou SJ Yuan J Wang C Zhao MH Chen M. Alternative complement pathway activation products in urine and kidneys of patients with ANCA-associated GN. Clin J Am Soc Nephrol (2013) 8:1884–91. 10.2215/cjn.02790313 PMC3817906 24115193

[36] Chen M Xing GQ Yu F Liu G Zhao MH. Complement deposition in renal histopathology of patients with ANCA-associated pauci-immune glomerulonephritis. Nephrol Dial Transpl (2009) 24:1247–52. 10.1093/ndt/gfn586 18940884

[37] Yang YZ Shi SF Chen YQ Chen M Yang YH Xie XF. Clinical features of IgA nephropathy with serum ANCA positivity: A retrospective case-control study. Clin Kidney J (2015) 8:482–8. 10.1093/ckj/sfv078 PMC4581394 26413270

[38] Bantis C Stangou M Schlaugat C Alexopoulos E Pantzaki A Memmos D. Is presence of ANCA in crescentic IgA nephropathy a coincidence or novel clinical entity? A case series. Am J Kidney Dis (2010) 55:259–68. 10.1053/j.ajkd.2009.09.031 20042261

[39] Chen A Lee K D’Agati VD Wei C Fu J Guan TJ. Bowman’s capsule provides a protective niche for podocytes from cytotoxic cd8+ t cells. J Clin Invest (2018) 128:3413–24. 10.1172/jci97879 PMC6063505 29985168

