# Supplementary material for: Glomerular Immune Deposition in MPO-ANCA Associated Glomerulonephritis Is Associated With Poor Renal Survival
Source: Front Immunol. 2021 Mar 25;12:625672. doi: 10.3389/fimmu.2021.625672 (PMC8027492; doi:10.3389/fimmu.2021.625672)
Supplement: Supplementary file 1 [file DataSheet_1.pdf]

Supplementary Information for

Glomerular immune deposition in MPO-ANCA associated glomerulonephritis is associated with poor renal survival

This file includes：

Supplemental Figure 1

Supplemental Table 1

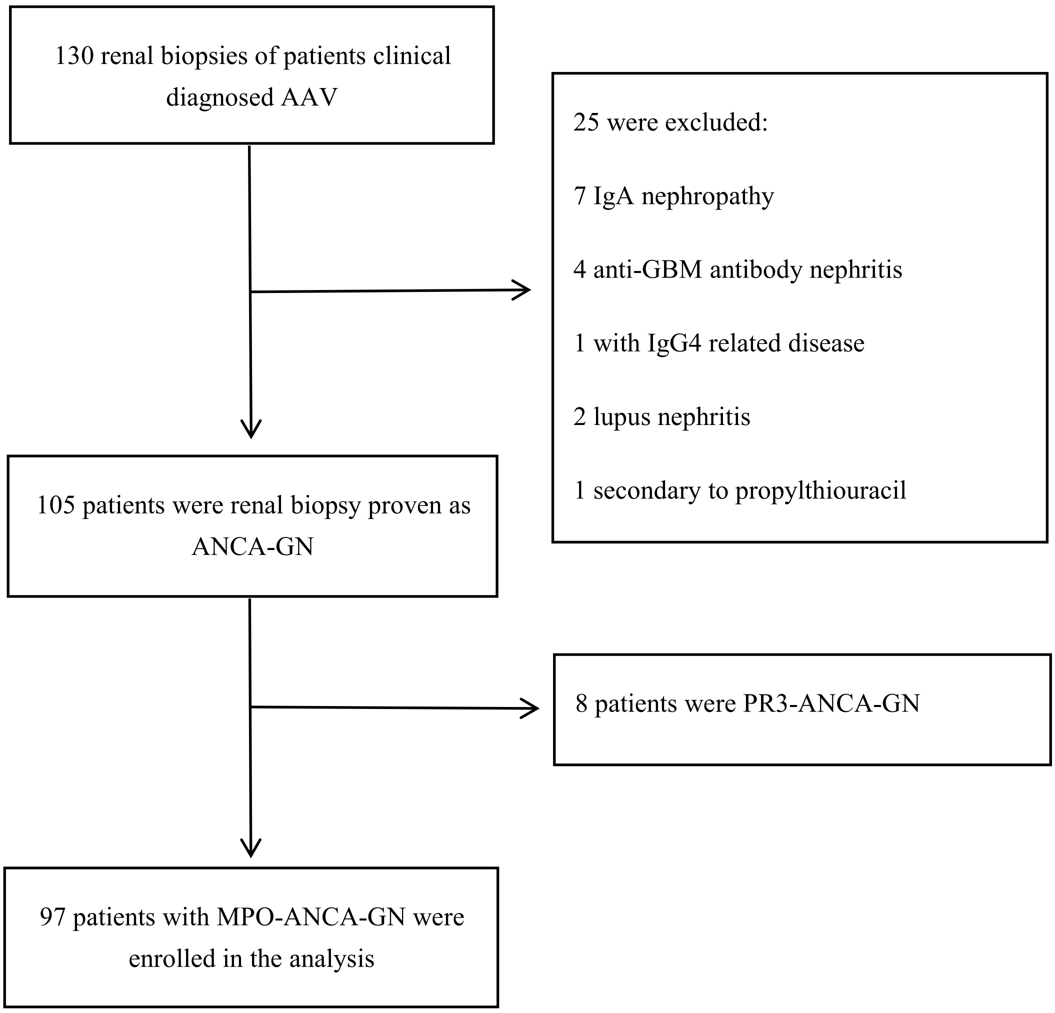

Figure S1 Flowchart of patients enrolled in the study

Table S1. Frequency of immune cells in MPO GN patients in the two groups

	PI[a] group (*N=51*)	IC[b] group (*N=39*)	Total (*N=90*)	*P*-value
Macrophages				
Glomerular (C/GCS[c]) (median, IQR)	1.06 (0.44, 3.78)	1.76 (0.40, 4.32)	1.57 (0.43, 4.08)	0.43
Periglomerular (C/GCS) (median, IQR)	1.88 (0.63, 5.00)	2.00 (0.62, 5.63)	2.00（0.63, 5.53）	0.82
Interstitial (C/HPF[d]) (median, IQR)	29.67 (13.33, 43.00)	32.67 (18.67, 46.33)	31.00 (14.75, 45.08)	0.18
Neutrophils				
Glomerular (C/GCS) (median, IQR)	0.36 (0, 1.31)	0.25 (0.00, 1.08)	0.33 (0.00, 1.17)	0.29
Periglomerular (C/GCS) (median, IQR)	0.00 (0.00, 0.69)	0.00 (0.00, 0.43)	0.00 (0.00, 0.47)	0.81
Interstitial (C/HPF) (median, IQR)	4.00 (1.33, 15.00)	4.33 (1.00, 10.67)	4.33 (1.25, 14.25)	0.79
T cells				
Glomerular (C/GCS) (median, IQR)	0.20 (0.00, 0.75)	0.15 (0.00, 0.83)	0.16 (0.00, 0.77)	0.65
Periglomerular (C/GCS) (median, IQR)	5.00 (1.10, 11.36)	3.78 (1.50, 8.78)	4.29 (1.18, 10.19)	0.47
Interstitial (C/HPF) (mean, SD)	56.05±29.98	56.39±44.73	56.20±36.87	0.97

[a]PI, pauci-immune; [b]IC, immune complex; [c]C/GCS, cells per glomerular cross-section; [d]C/HPF, cells per high-powered field.
